# Surgical treatment of locally advanced right colon cancer invading neighboring organs

**DOI:** 10.3389/fmed.2022.1044163

**Published:** 2023-01-13

**Authors:** HyokJu Ri, HaoNan Kang, ZhaoHui Xu, ZeZhong Gong, HyonSu Jo, Boureima Hamidou Amadou, Yang Xu, YanYing Ren, WanJi Zhu, Xin Chen

**Affiliations:** ^1^Department of Hernia and Colorectal Surgery, The Second Hospital of Dalian Medical University, Dalian, China; ^2^Department of Colorectal Surgery, The Hospital of Pyongyang Medical College, Pyongyang, Democratic People’s Republic of Korea

**Keywords:** advanced colon cancer, en bloc resection, pancreaticoduodenectomy, hemicolectomy, multivisceral resection

## Abstract

**Purpose:**

Invasion of the pancreas and/or duodenum with/without neighboring organs by locally advanced right colon cancer (LARCC) is a very rare clinical phenomenon that is difficult to manage. The purpose of this review is to suggest the most reasonable surgical approach for primary right colon cancer invading neighboring organs such as the pancreas and/or duodenum.

**Methods:**

An extensive systematic research was conducted in PubMed, Medline, Embase, Scopus, and the Cochrane Central Register of Controlled Trials (CENTRAL) using the MeSH terms and keywords. Data were extracted from the patients who underwent en bloc resection and local resection with right hemicolectomy (RHC), the analysis was performed with the survival rate as the outcome parameters.

**Results:**

As a result of the analysis of 117 patient data with locally advanced colon cancer (LACC) (73 for males, 39 for females) aged 25–85 years old from 11 articles between 2008 and 2021, the survival rate of en bloc resection was 72% with invasion of the duodenum, 71.43% with invasion of the pancreas, 55.56% with simultaneous invasion of the duodenum and pancreas, and 57.9% with invasion of neighboring organs with/without invasion of duodenum and/or pancreas. These survival results were higher than with local resection of the affected organ plus RHC.

**Conclusion:**

When the LARCC has invaded neighboring organs, particularly when duodenum or pancreas are invaded simultaneously or individually, en bloc resection is a reasonable option to increase patient survival after surgery.

## Highlights

- Invasion of the pancreas and/or duodenum with/without neighboring organs by locally advanced right colon cancer (LARCC) is a very rare clinical phenomenon that is difficult to manage.
- A few studies have attempted to find the reasonable surgical approach to get high survival focusing on en bloc resection when the LARCC invaded neighboring organs.

- The en bloc resection is the gold standard surgical options for LARCC invading neighboring organs when there is no distant metastasis.
- This is important to raise awareness among clinicians and researchers to focus on en bloc resection, to improve patient survivals of LARCC with invading neighboring organs.

## 1. Introduction

The colorectal cancer is the third most common cancer in the world, accounting for more than a third of all cancer cases worldwide, and the mortality rate is usually high (1, 2).

In general, surgery is the first choice for colon cancer, and non-radical resection and blunt mobilization of the attached organs is associated with tumor recurrence and prognosis, particularly when the colon cancer invades neighboring organs, which is defined as “locally advanced colon cancer (LACC)” (3–6). The RCC also occasionally invades the pancreas and/or duodenum in the clinics, and this can cause troubles in the operation because these organs are attached due to an inflammatory or oncologic reaction (7–9).

When LACC invades the several neighboring organs with pancreas and/or duodenum, the first option to consider is performing the multi-organic or extended resection for the more achieving tumor negative margin of the resection (10–12).

Therefore, the en bloc resection is the gold standard surgical options for LACC invading neighboring organs when there is no distant metastasis. The goal of surgical resection of primary colon cancer is complete removal of the tumor, the major vascular pedicles, and the lymphatic drainage basin of the affected colonic segment, and the en bloc resection of contiguous structures is indicated if there is attachment or infiltration of the tumor into a potentially resectable organ or structure (13, 14).

An understanding of these issues may prove important to prolong survival, and surgical options for invading neighboring organs of RCC have been continuously explored over the past several decades (12, 15–23). Unfortunately, their outcomes have the limitations coming from the lack of study samples and designs, and despite of the perfect procedures in the operation, it is still remained unclear which operation is the best options for locally advanced RCC (LARCC) (8, 13, 17, 24–28).

The aim of this study is to provide comprehensive knowledge through the systematic review of fundamental literatures and to find a reasonable surgical approach for LARCC with invading neighboring organs.

## 2. Materials and methods

### 2.1. Research design and criteria

This systematic review was conducted in accordance with the preferred reporting items for systemic review and meta-analysis (PRISMA) statement. The electronic records provided a wealth of rich data and the large sample size required for this study, and the basic data were generated using the appropriate inclusion and exclusion criteria.

#### 2.1.1. Inclusion criteria

The randomized controlled trials and non-randomized controlled trials of surgical treatment of LARCC with invasion of adjacent organs such as pancreas and/or duodenum were included in this study.

#### 2.1.2. Exclusion criteria

Low quality studies (no detailed explanation pre-operation and surgical process) and studies with poor outcomes (no more than two outcome parameters) were excluded from this study.

#### 2.1.3. Patient information

The adult patients (aged 25–85 years old) with LARCC invading neighboring organs without gender specification.

### 2.2. Systematic fundamental literature search

An extensive systematic search was conducted in PubMed, Medline, Embase, Scopus, and the Cochrane Central Register of Controlled Trials (CENTRAL) using the MeSH terms and keywords: (([All Fields] = (locally advanced*)) AND [All Fields] = (right*) AND [All Fields] = (colon* OR colonic*)) AND [All Fields] = (cancer* OR tumor* OR carcinoma*) AND [All Fields] = (invading* OR affecting*) AND ([All Fields] = (pancreas) OR [All Fields] = (duodenum) OR [All Fields] = (multivisceral*))) OR ((([All Fields] = (right*) AND [All Fields] = (hemicolectomy)) OR [All Fields] = (pancreatoduodenectomy)).

**FIGURE 1:**
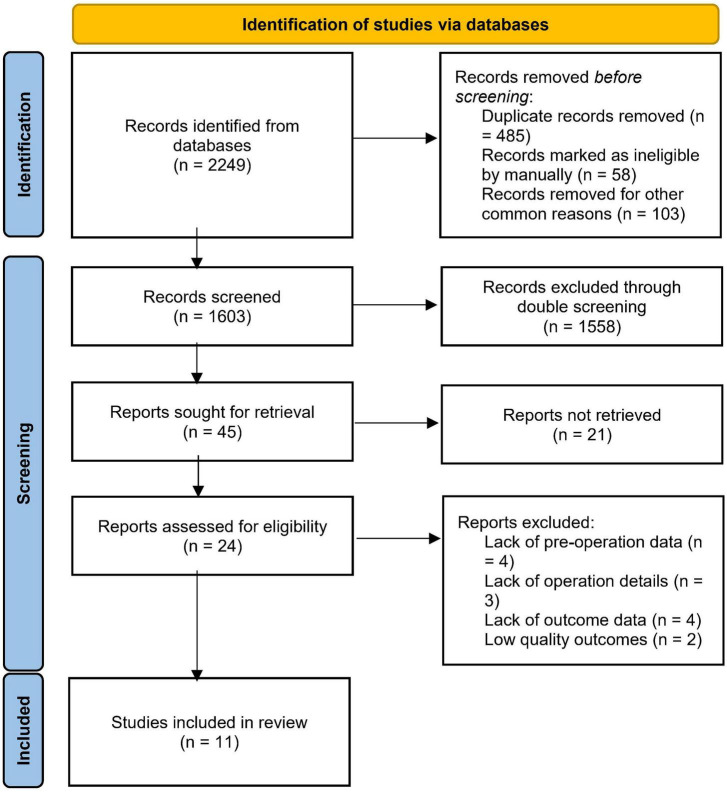
Preferred reporting items for systemic review and meta-analysis (PRISMA) flow chart for literature search.

This study was conducted in April 2022 and the search process was performed manually. After collecting records, the three researchers consulted on the database and the very early pre-1950s studies and duplicates were first excluded, then the records were independently sourced according to the inclusion and exclusion criteria by researchers and finally merged into the core database (Figure 1).

### 2.3. Data extraction

The data was extracted based on the Cochrane Consumers and Communication Review Group’s data extraction template.

### 2.4. Data analysis

We divided LARCC cases into invasion of the duodenum alone, invasion of the pancreas alone, invasion of the duodenum and pancreas simultaneously, or invasion of the pancreas and/or duodenum with other adjacent organs. And the analysis was performed with the survival rate as the outcome parameter.

## 3. Results

### 3.1. The characteristics of included studies

Between 2008 and 2021, 11 studies involving 117 patients with LARCC invasion of neighboring organs were collected from the database search (Table 1). Among them, 73 patients (62.4%) were men and 39 patients (33.3%) were women, and the last 5 patients (4.3%) were unreported their gender information. All patients were ranged between 25 and 85 years old, and the primary tumors were located in the right colon, particularly in the hepatic flexion.

**TABLE 1:** The characteristics of included studies.

No.	First author	Country	Published year	Number of patients	Diagnosis (with invading neighboring organs)	Operation methods
1	A. Saiura	Japan	2008	12	RCC with invading D and/or P	Eb bloc PD + RHC
2	E. T. Kimchi	U.S.	2009	14	RCC with invading D, P, and M	PPPD, PD, RHC, RN
3	W. S. Lee	South Korea	2009	9	RCC with invading D and/or P	Eb bloc PD + RHC, PPPD + RHC
4	S. R. Costa	Brazil	2010	5	RCC with invading D and/or P and/or S and/or K	Eb bloc PD + RHC, PPPD + RHC with GDP or RN
5	J. Zhang	China	2013	14	RCC with invading D and/or P	Eb bloc PD + RHC with RN or PH
6	Y. Kaneda	Japan	2017	5	RCC with invading D and/or P and/or S and/or SMV	PD + RHC + SMVR
7	C. Agalar	Turkey	2017	5	RCC with invading D and/or P	Eb bloc PD + RHC
8	X. L. Yan	China	2021	19	RCC with invading D and/or P and/or S and/or L and/or SMV	Eb bloc PD + RHC with SMVR if necessary
9	N. Cojocari	Romania	2021	17	RCC with invading D and/or P	Eb bloc PD + RHC, Du + RHC, Pa + RHC
10	J. B. Chen	China	2021	11	RCC with invading D and/or P	PD + RHC, PPPD + RHC
11	S. S. Uludag	Turkey	2021	6	RCC with invading D and/or P	Eb bloc PD + RHC

RCC, right colon cancer; D, duodenum; P, pancreas; M, mesentery; S, stomach; K, kidney; L, liver; SMV, superior mesenteric vein; PPPD, pylorus preserving pancreaticoduodenectomy; PD, pancreaticoduodenectomy; Du, duodenal resection; Pa, pancreatectomy; RHC, right hemicolectomy; GDP, gastroduodenopancreatectomy; RN, right nephrectomy; PH, partial hepatectomy; SMVR, superior mesenteric vein resection.

### 3.2. Comparison of classic pancreaticoduodenectomy and en bloc resection with right hemicolectomy when RCC invaded duodenum

Patient information, treatments, and outcome data are presented in Table 2. A total of 35 out of 117 patients were diagnosed RCC with invading duodenum, and two surgical methods including classic pylorus preserving pancreaticoduodenectomy (PPPD) and en bloc resection with right hemicolectomy (RHC) were performed. Of 10 patients who performed PPPD and local duodenal resection plus RHC, 5 patients died, including 2 of them with recurrences, while 5 patients are alive, of which 1 had recurrence. The survival rate was 50%. Of the 25 cases of en bloc resection, seven patients died from recurrence. The remaining 18 patients were alive and did not relapse. The survival rate was 72%.

**TABLE 2:** The comparison of local pancreaticoduodenectomy and en bloc PD resection with RHC when right colon cancer (RCC) invaded duodenum.

No.	Gender	Age	Invading location	T stage	N stage	M stage	Operation method	Complication	Status	Disease free (month)	Recurrence
1	M	75	D + RC	4	0	0	Du + RHC	No	Dead	11	Yes
2	M	62	D + RC	4	2	0	Du + RHC	N/A	Dead	39	Yes
3	F	76	D + RC	4	0	0	Du + RHC	N/A	Alive	24	No
4	M	66	D + RC	4	0	0	Du + RHC	N/A	Alive	28	No
5	M	55	D + RC	4	2	0	Du + RHC	N/A	Dead	11	No
6	M	52	D + RC	4	0	0	Du + RHC	N/A	Dead	N/A	N/A
7	M	66	D + RC	N/A	N/A	N/A	Du + RHC	N/A	Alive	6	No
8	M	68	D + RC	N/A	N/A	N/A	Du + RHC	N/A	Alive	43	No
9	M	64	D + TVC	4	1	0	PPPD + RHC	No	Alive	11	Yes
10	M	73	D + RC	N/A	N/A	N/A	PPPD + RHC	N/A	Dead	N/A	N/A
11	M	50	D + TVC	N/A	N/A	N/A	En bloc PD + RHC	No	Alive	41	No
12	M	83	D + RC	4	0	0	En bloc PD + RHC	PF, DGE	Alive	12	No
13	M	46	D + HFC	4	1	0	En bloc PD + RHC	PF	Alive	60	No
14	F	76	D + HFC	4	1	0	En bloc PD + RHC	PF, DGE	Dead	10	Yes
15	F	48	D + ASC	4	0	0	En bloc PD + RHC	N/A	Alive	276	No
16	M	58	D + HFC	4	1	0	En bloc PD + RHC	DGE	Alive	18	No
17	F	73	D + TVC	4	0	1	En bloc PD + RHC	PF	Alive	77	No
18	M	60	D + HFC	N/A	N/A	N/A	En bloc PD + RHC	N/A	Dead	16	Yes
19	M	75	D + HFC	N/A	N/A	N/A	En bloc PD + RHC	PF	Dead	36	Yes
20	M	62	D + ASC	N/A	N/A	N/A	En bloc PD + RHC	PF	Dead	9	Yes
21	M	36	D + HFC	N/A	N/A	N/A	En bloc PD + RHC	No	Alive	69	No
22	F	65	D + HFC	N/A	N/A	N/A	En bloc PD + RHC	PF	Alive	63	No
23	M	45	D + HFC	N/A	N/A	N/A	En bloc PD + RHC	PF, IAA	Dead	9	Yes
24	M	73	D + HFC	N/A	N/A	N/A	En bloc PD + RHC	BF	Dead	41	Yes
25	M	35	D + HFC	N/A	N/A	N/A	En bloc PD + RHC	PF	Alive	48	No
26	F	46	D + HFC	N/A	N/A	N/A	En bloc PD + RHC	BF, IAA	Alive	41	No
27	M	63	D + HFC	N/A	N/A	N/A	En bloc PD + RHC	No	Alive	23	No
28	M	64	D + HFC	N/A	N/A	N/A	En bloc PD + RHC	No	Alive	17	No
29	F	66	D + HFC	N/A	N/A	N/A	En bloc PD + RHC	PF	Alive	8	No
30	F	65	D + HFC	N/A	N/A	N/A	En bloc PD + RHC	No	Alive	5	No
31	M	62	D + RC	4	1	0	En bloc PD + RHC + PG	No	Alive	180	No
32	F	77	D + HFC	N/A	N/A	0	En bloc PD + RHC	N/A	Dead	168	Yes
33	F	51	D + HFC	N/A	N/A	0	En bloc PD + RHC	N/A	Alive	216	No
34	M	55	D + HFC	N/A	N/A	0	En bloc PD + RHC	N/A	Alive	60	No
35	M	61	D + HFC	N/A	N/A	1	En bloc PD + RHC	PF	Alive	154	No

M, male; F, female; D, duodenum; RC, right colon; ASC, ascending colon; TVC, transverse colon; RHC, right hemicolectomy; PG, partial gastrectomy; BF, biliary fistula; PF, pancreatic fistula; IAA, intra-abdominal abscess; HFC, hepatic flex colon; DGE, delayed gastric emptying; PPPD, pylorus preserving pancreaticoduodenectomy; Du, duodenal resection; N/A, not reported.

### 3.3. Comparison of classic pancreaticoduodenectomy and en bloc resection with right hemicolectomy when RCC invaded pancreas

All information has been listed in Table 3. RCC with invading pancreas was diagnosed in 14 of 117 patients, and surgical approaches such as local pancreatectomy, classic PPPD, and en bloc resection with RHC were performed. Of seven patients who performed local pancreatectomy or PPPD, four patients died, including 3 of them with recurrences, while three patients were alive and none of them had recurrences. The survival rate was 42.86%. Of the seven cases that underwent en bloc resection, two patients died due to recurrence. The remaining five patients were still alive and had no relapse. The survival rate was 71.43%.

**TABLE 3:** The comparison of local pancreaticoduodenectomy and en bloc PD resection with RHC when right colon cancer (RCC) invaded pancreas.

No.	Gender	Age	Invading location	T stage	N stage	M stage	Operation method	Complication	Status	Disease free (month)	Recurrence
1	F	63	P + RC	2	1	0	PPPD + RHC	No	Alive	19	No
2	M	67	P + RC	1	1	0	PPPD + RHC	No	Dead	22	Yes
3	M	85	P + RC	2	1	0	PPPD + RHC	No	Dead	16	N/A
4	F	64	P + RC	3	1	0	PPPD + RHC	No	Dead	13	Yes
5	F	65	P + RC	1	0	0	PPPD + RHC	N/A	Alive	11	No
6	M	71	P + HFC	4	1	0	PPPD + RHC	DGE	Alive	27	No
7	F	74	P + RC	4	1	0	Pa + RHC	N/A	Dead	7	Yes
8	M	64	P + RC	3	1	0	En bloc PD + RHC	DGE	Alive	9	No
9	F	83	P + RC	4	1	0	En bloc PD + RHC	SSI	Dead	5	Yes
10	F	76	P + RC	3	1	0	En bloc PD + RHC	POB	Dead	20	Yes
11	M	39	P + HFC	4	0	0	En bloc PD + RHC	PF	Alive	48	No
12	M	69	P + HFC	4	2	0	En bloc PD + RHC	IAA	Alive	N/A	No
13	M	25	P + RC	4	0	0	En bloc PD + RHC	No	Alive	96	No
14	M	62	P + HFC	N/A	N/A	0	En bloc PD + RHC	No	Alive	42	No

M, male; F, female; P, pancreas; RC, right colon; Pa, pancreatectomy; PPPD, pylorus preserving pancreaticoduodenectomy; PD, pancreaticoduodenectomy; RHC, right hemicolectomy; SSI, surgery site infection; POB, post-operation bleeding; DGE, delayed gastric emptying; HFC, hepatic flex colon; PF, pancreatic fistula; IAA, intra-abdominal abscess; N/A, not reported.

### 3.4. Comparison of classic pancreaticoduodenectomy and en bloc resection with right hemicolectomy when RCC invaded duodenum and pancreas simultaneously

All patient, surgery and outcome information are presented in Table 4. Eighteen of 117 patients were diagnosed with RCC invading the duodenum and pancreas simultaneously and all underwent en bloc resection with RHC. A total of 18 patients who performed en bloc resection, 8 patients died, including 3 with recurrence and 5 without information of recurrence, and the remaining 10 patients are alive and all had no recurrences. The survival rate was 55.56%.

**TABLE 4:** The comparison of local pancreaticoduodenectomy and en bloc PD resection with RHC when right colon cancer (RCC) invaded pancreas and duodenum.

No.	Gender	Age	Invading location	T stage	N stage	M stage	Operation method	Complication	Status	Disease free (month)	Recurrence
1	F	62	D + P + HFC	4	1	0	En bloc PD + RHC	BF, DGE	Dead	12	Yes
2	F	45	D + P + HFC	4	0	0	En bloc PD + RHC	None	Alive	24	No
3	M	47	D + P + ASC	4	0	0	En bloc PD + RHC	PF, IAA	Dead	N/A	N/A
4	M	54	D + P + HFC	4	0	0	En bloc PD + RHC	PF, DGE	Alive	12	No
5	M	70	D + P + ASC	4	0	0	En bloc PD + RHC	PF, DGE	Alive	95	No
6	M	48	D + P + ASC	N/A	N/A	N/A	En bloc PD + RHC	PF, IAA	Dead	25	Yes
7	M	65	D + P + HFC	N/A	N/A	N/A	En bloc PD + RHC	PF	Alive	47	No
8	F	42	D + P + HFC	N/A	N/A	N/A	En bloc PD + RHC	PF	Alive	20	No
9	M	61	D + P + RC	4	1	0	En bloc PD + RHC	N/A	Dead	N/A	N/A
10	M	52	D + P + RC	4	1	0	En bloc PD + RHC	N/A	Dead	12	Yes
11	M	65	D + P + RC	4	1	0	En bloc PD + RHC	N/A	Alive	24	No
12	M	69	D + P + RC	4	1	0	En bloc PD + RHC	N/A	Alive	31	No
13	F	59	D + P + HFC	N/A	1	N/A	En bloc PD + RHC	DF	Dead	1	N/A
14	M	49	D + P + HFC	N/A	1	N/A	En bloc PD + RHC	N/A	Dead	39	N/A
15	M	68	D + P + HFC	N/A	1	N/A	En bloc PD + RHC	N/A	Dead	10	N/A
16	M	65	D + P + RC	N/A	N/A	N/A	En bloc PD + RHC	N/A	Alive	40	No
17	M	57	D + P + RC	N/A	N/A	N/A	En bloc PD + RHC	N/A	Alive	31	No
18	M	73	D + P + RC	N/A	N/A	N/A	En bloc PD + RHC	N/A	Alive	26	No

M, male; F, female; D, duodenum; P, pancreas; RC, right colon; ASC, ascending colon; HFC, hepatic flex colon; PD, pancreaticoduodenectomy; RHC, right hemicolectomy; BF, biliary fistula; DF, duodenum fistula; PF, pancreatic fistula; DGE, delayed gastric emptying; IAA, intra-abdominal abscess; N/A, not reported.

### 3.5. En bloc resection with right hemicolectomy when RCC invaded pancreas and/or duodenum with other neighboring organs

All patient, surgery and outcome information are presented in Table 5. RCC invading the pancreas and/or duodenum with other neighboring organs was diagnosed in 24 of 117 patients, and 19 of them underwent en bloc resection with RHC and 5 patients underwent PPPD with RHC. In addition, local resection of penetrating neighboring organs was performed in all cases. A total of 8 patients out of 19 patients with en bloc resection died, while two out of 5 patients with PPPD died. The survival rate of en bloc resection was is 57.9%.

**TABLE 5:** En bloc PD with RHC when right colon cancer (RCC) invaded pancreas and/or duodenum with other neighboring organs.

No	Gender	Age	Invading location	T stage	N stage	M stage	Operation method	Complication	Status	Disease free (month)	Recurrence
1	F	63	P + RC + Mes	2	1	0	PPPD + RHC	None	Alive	19	N/A
2	M	67	P + RC + Mes	1	1	0	En bloc PD + RHC	None	Dead	22	N/A
3	M	85	P + RC + Mes	2	1	0	En bloc PD + RHC	None	Dead	16	N/A
4	F	64	P + RC + Mes	3	1	0	En bloc PD + RHC	N/A	Dead	13	N/A
5	F	76	P + RC + Mes	3	1	0	PPPD + RHC	POB	Dead	20	N/A
6	F	65	P + RC + Mes	1	0	0	PPPD + RHC + SMVR	None	Alive	11	No
7	M	64	P + RC + Mes	3	1	0	En bloc PD + RHC	DGE	Alive	9	No
8	M	64	D + RC + Mes	4	1	0	PPPD + RHC	None	Dead	11	Yes
9	F	56	D + P + K + HFC	4	0	0	En bloc PD + RHC	PF, DGE	Alive	63	No
10	M	46	D + Gb + L + HFC	4	2	0	En bloc PD + RHC + PH	DGE	Alive	49	No
11	M	48	D + L + HFC	4	0	0	En bloc PD + RHC	PF, DGE, IAA	Alive	4	N/A
12	F	54	D + K + ASC	4	2	0	En bloc PD + RHC + Ne	None	Alive	13	No
13	F	74	D + P + S + ASC	4	1	1	En bloc PD + RHC + SMVR	PF	Dead	11	Yes
14	F	57	D + P + S + SMV + HFC	4	0	0	En bloc PD + RHC + PG + SMVR	PF	Alive	85	No
15	M	47	D + P + SMV + ASC	4	1	1	En bloc PD + RHC + SMVR	PF	Dead	11	Yes
16	F	44	D + P + SMV + HFC	N/A	N/A	N/A	En bloc PD + RHC + SMVR	None	Alive	112	No
17	M	48	D + L + HFC	N/A	N/A	N/A	En bloc PD + RHC + PH	PF, IAA	Alive	103	No
18	M	54	D + L + HFC	N/A	N/A	N/A	En bloc PD + RHC	Ileus	Alive	30	No
19	M	65	D + P + K + RC	4	2	0	En bloc PD + RHC + Ne	N/A	Dead	N/A	N/A
20	F	64	D + P + K + RC	4	0	0	PPPD + RHC + Ne	N/A	Alive	36	N/A
21	M	50	P + S + HFC	N/A	0	N/A	En bloc PD + RHC	GF	Dead	25	N/A
22	M	34	P + S + TVC	N/A	0	N/A	En bloc PD + RHC	GF	Alive	75	N/A
23	M	69	D + H + ASC	N/A	1	N/A	En bloc PD + RHC	DF	Dead	15	N/A
24	F	62	D + S + TVC	N/A	0	N/A	En bloc PD + RHC	None	Alive	3	N/A

M, male; F, female; P, pancreas; K, kidney; S, stomach; Gb, gallbladder; L, liver; RC, right colon; Mes, mesentery; ASC, ascending colon; RHC, right hemicolectomy; TVC, transverse colon; PPPD, pylorus preserving pancreaticoduodenectomy; PH, partial hepatectomy; PG, partial gastrectomy; POB, post-operation bleeding; DGE, delayed gastric emptying; DF, duodenum fistula; GF, gastric fistula; SMVR, superior mesenteric vein resection; PF, pancreatic fistula; IAA, intra-abdominal abscess; Ne, nephrectomy; N/A, not reported.

## 4. Discussion

Right colon cancer is usually treated with a RHC. This surgical treatment has solved many oncological and radical resection problems and is now widely used in clinical practice (29–31). However, if the RCC invades the duodenum and/or pancreas, or if it invades other neighboring organs simultaneously, the treatment is relatively difficult and the post-operative mortality rate is relatively high (8, 15, 28, 32–34). LACC in adjacent organs is a rare phenomenon, recently 5.2–23.6% of all colorectal cancers invade or attach to adjacent organs at this time of presentation (35). This problem had received the substantial interests of colorectal surgeons and they finally produced the significant operation procedure, the RHC with pancreatoduodenectomy which was defined as en bloc resection. This surgical method is considered to be quite difficult surgically in general, and there are relatively many complications including post-operative pancreas and/or gallbladder fistula, and post-operative patient management is relatively difficult and complex while the advantage of this en bloc resection is relatively radical and increases the survival rate of the patient after surgery (3, 15, 36).

The en bloc resection approach was first introduced in 1953 (37). At that time, the post-operative mortality and recurrence rate, and the incidence of post-operative complications were very high due to insufficient surgical equipment and inexperienced surgical technique, especially incomplete resection (38–41). With the development of numerous medical facilities, from laparotomy through laparoscopy to robotic surgery, and the rapid developing of medical science and technology have broken the difficulty of the en bloc resection, greatly increased the possibility of surgery, and lowered the incidence of post-operative complications (42–50). Currently, when RCC has invaded the duodenum and/or pancreas, this en bloc resection is the most reasonable surgical treatment option and several studies reported the long-term outcomes of this procedure (51–57).

In Saiura et al. (19) reported that pancreaticoduodenectomy for the advanced RCC invading the duodenum and/or pancreas was beneficial with a 5-year survival rate of 55%. In Costa et al. (58) reported that pancreatoduodenectomy plus RHC for LARCC could provide the long-term survival rates, while there were sample size limitations in five T4 patients. In Cojocari et al. (20) reported that four patients underwent RHC with duodenectomy and they had no recurrence at 11–39 months, and this surgical method could be a good choice for right-sided colon cancer invading neighboring organs.

In Curley et al. (51) reported the resection for cure of carcinoma of the colon directly invading the duodenum or pancreatic head, and the survival rate with en bloc resection is relatively high than local resection, especially in invading pancreatic head cases. They concluded long-term survival could be achieved by en bloc resection in patients with locally advanced carcinoma of the colon involving the duodenum or pancreatic head. In Sasson et al. (42) reported the en bloc resection for locally advanced pancreatic cancer. In their study, 13 of 116 patients required partial colectomy to completely remove the lesion because extensive involvement of the mesentery of the transverse and right colon resulted in significant shortening of the mesentery. However, they could not find any differences in outcomes for patients with locally advanced cancer requiring en bloc resection compared to patients with standard pancreatectomy, and only suggested that en bloc resection involving surrounding structures to completely remove all macroscopic disease in selected patients with locally advanced disease might be beneficial, particularly when combined with pre-operative chemoradiotherapy. The results like these can be found in papers published by other research groups (59–64).

In our study, the survival rate with en bloc resection was relatively higher than with RHC plus local duodenectomy, RHC plus local pancreatectomy, and RHC plus PPPD. This result was consistent with previous reports in several studies and it was because this operation could achieve the tumor clearance in patients with an adherent but not penetrating right colon carcinoma when RCC invades duodenum or pancreas alone or simultaneously (16, 18, 23, 65–67).

As we have already discussed, RCC is more advanced and the most commonly affected organs are the duodenum and pancreas. In some cases, however, abdominal organs such as the stomach, liver, gallbladder, and kidneys, etc., are affected singly or in combination, and there are also cases of invasion of the peritoneum and/or mesentery (68–71). In these cases, the partial excision of the affected organ and the RHC are performed simultaneously (16, 20, 23, 72). Unfortunately, in these cases there are many post-operative complications with a high rate of recurrence and a low survival rate. In combination with metastatic comorbidities, the prognosis is not so good (73, 74).

We also summarized that en bloc resection with RHC when RCC invaded pancreas and/or duodenum with other neighboring organs, and the survival rate was is 57.9%. This result still tells us that the mortality in these cases is relatively high and that the advanced study is needed to increase the patient’s survival.

This research has some shortcomings. It is a method of collecting English-only documents within the scope of collecting data, which does not guarantee the wide range of the research scope. It is revealed that en bloc resection is a very beneficial option only in terms of the surgical method without revealing the factors that have a great influence on the survival rate of post-operative patients, such as post-operative chemotherapy and lymph node-negative status, which were announced in some studies. In addition, the time interval in this study is relatively long, from 2008 to 2021. This may lead to a possible bias in the results of the study, but in practice, LARCC invading neighboring organs is very rare and there is insufficient data to avoid this bias by shortening the time interval. In this regard, we hope that readers will read the study results with caution. In the future, we plan to further supplement and deepen the related contents in future research.

## 5. Conclusion

This systematic review concludes that when LARCC has invaded neighboring organs, en bloc resection is a reasonable option to prolong patient survival after surgery.

## Data availability statement

The raw data supporting the conclusions of this article will be made available by the authors, without undue reservation.

## Author contributions

HR, XC, and HK: conceptualization. ZX, ZG, HJ, and BA: investigation. YX, YR, and WZ: data curation. HR, HK, and XC: writing—original draft preparation. ZX, ZG, HJ, YX, YR, and WZ: writing—review and editing. HR, XC, and BA: supervision and project administration. All authors have read and agreed to the submitted version of the manuscript.
